# Developmental Profile of Executive Functioning in School-Age Children From Northeast Brazil

**DOI:** 10.3389/fpsyg.2020.596075

**Published:** 2021-01-14

**Authors:** Amanda Guerra, Izabel Hazin, Yasmin Guerra, Jean-Luc Roulin, Didier Le Gall, Arnaud Roy

**Affiliations:** ^1^Programa de Pós-Graduação de Psicologia, Universidade Federal do Rio Grande do Norte, Natal, Brazil; ^2^Laboratoire de psychologie des Pays de la Loire (EA 4638), Université d’Angers, Angers, France; ^3^Departamento de Psicologia, Universidade Federal do Rio Grande do Norte, Natal, Brazil; ^4^Laboratoire de Psychologie et NeuroCognition (UMR 5105), Université de Savoie Mont Blanc, Chambéry, France; ^5^Centre Référent des Troubles d’Apprentissage, Centre de Compétence Nantais de Neurofibromatose, Hôpital Femme-Enfant-Adolescent, CHU de Nantes, Nantes, France

**Keywords:** socioeconomic status, development, child, culture, Neuropsychology

## Abstract

The development of executive functions (EF) is recognizably correlated to culture, contextual and social factors. However, studies considering all the basic EF are still scarce in Brazil, most notably in the Northeast region, which is known for its social inequality and economic gap. This study aimed to analyze the developmental trajectories and structure of four EF, namely inhibition, flexibility, working memory and planning. In addition, the potential effects of socioeconomic status (SES) and gender were examined. The sample included 230 Brazilian children between 7-12 years old, homogeneously distributed by age, gender and type of school. The EF were assessed through the Brazilian version of the Child Executive Functions Battery (CEF-B). A global effect of age was found for most of the EF measures evaluated. Gender effect was mostly non-significant, except for 4 of the 12 tasks. There was a significant SES effect on 8 tasks, all in favor of private school children. Exploratory factorial and correlation analysis showed a 4-factor EF structure, corroborating the theoretical distribution considered in the CEF-B. A developmental progression is evident in the results for all of the EF measures evaluated. While gender had little influence on EF, SES seems to significantly impact the development of EF. As normative data are still lacking in Northeast Brazil, this study may help to understand EF development trajectories and provide tools for neuropsychological evaluation.

## Introduction

Executive functions (EF) comprise a set of superior cognitive skills that allow the subject to engage in goal-oriented behaviors (Luria, 1966). These skills are considered as a predictor for success in various aspects of life and are essential for guiding and regulating intellectual, emotional and social abilities (Diamond, 2013; Zelazo, 2015). Especially in children, EF have been pointed out as predictors of academic success. In several studies, the performance in executive tests is more correlated with school success than the performance in intelligence tests during the first years of school (Shaul and Schwartz, 2014; Follmer, 2017).

Most of classical studies recognize that EF consist of three core skills that are categorized differently according to certain authors and theoretical models. There seems to be a relative consensus regarding the categorization of the inhibition component as one of these three basic factors (e.g., Miyake et al., 2000; Lehto et al., 2003; Diamond, 2013; Friedman and Miyake, 2017). The definition of the two other components seems to vary between working memory (Anderson, 2002; Lehto et al., 2003; Brocki and Bohlin, 2004; Diamond, 2013) vs updating (Miyake et al., 2000; Agostino et al., 2010), and shifting (Miyake et al., 2000; Lehto et al., 2003; Agostino et al., 2010) vs cognitive flexibility (Anderson, 2002; Diamond, 2013) both in adult and child-centered models (Morra et al., 2018). In fact, the theoretical distinction between these components is far from being consensual in the literature. Similarly, the debate on the so-called complex executive functions is also inconsistent and diverse. Diamond (2013) considers that basic EF (described in her model as inhibition, WM and flexibility) are implied in the operation of higher-level EF such as planning, reasoning and problem solving. Among these complex components, planning is often derived from EF studies with clinical background (Gioia et al., 2000; Anderson, 2002; Roy et al., 2010). Despite basic and complex components are considered an independent construct through a theoretical perspective, they are strongly interrelated (Miyake et al., 2000; Lehto et al., 2003; Diamond, 2013).

Executive functions start operating since the first years of life, but would follow a progressive developmental trajectory and reach a late functional maturity at approximately 25 to 30 years of age (Lebel et al., 2008; Tamnes et al., 2010). This long trajectory would be characterized by spurts or peaks in development and by different organizational and structural transformations (Anderson, 2002). Factorial analysis studies in preschoolers have shown that EF are still relatively undifferentiated until approximately the age of 5 (see Lee et al., 2013). In fact, studies conducted with 3-year-olds have described an EF structure comprising a single latent variable (Willoughby et al., 2010, 2012). Studies carried out with 4 and 5 years old children support both a unitary model (Shing et al., 2010; Fuhs and Day, 2011) and a two-factor model (Miller et al., 2012; Lee et al., 2013; van der Ven et al., 2013; Usai et al., 2014; Panesi and Morra, 2020). In contrast, studies seem to agree that after 6 years, EF would gradually specialize, approaching a multifactorial structure such as identified in adults (Brocki and Bohlin, 2004; Huizinga et al., 2006).

These developmental studies have also reported the influence of other demographic variables on EF, such as gender. In the majority of researches, gender effect on executive performances has proven to be non-significant (Anderson, 2002; Brocki and Bohlin, 2004; Huizinga et al., 2006; Lee et al., 2013; Xu et al., 2013). However, some North American studies showed significant differences in favor of boys (Halpern, 2012) while studies in Mexico and Colombia were in favor of girls (Ardila et al., 2005). In these cases, gender effect seems to vary according to the tasks used and, more broadly, to cultural aspects (Roukoz et al., 2018).

In fact, the role of social, cultural and contextual factors in the emergence of EF in children has been increasingly recognized (Sbicigo et al., 2013; Farah, 2017; Lawson et al., 2017). Several constructs are used as correlated measures to evaluate the impact of environmental context in executive development. Socioeconomic status (SES) is currently considered one of the most used factor to assess the impact of different life contexts on EF development. However, SES is a challenging construct to measure because it comprises multiple social and economic variables related with educational achievement, health, and psychological well-being (Farah, 2017). Different indicators such as parents’ education and profession, family income, type of school (private or public) or a combination of these factors recognizably impact the development of EF, especially WM, selective attention, inhibitory control and cognitive flexibility (Noble et al., 2015; Johnson et al., 2016; Ursache and Noble, 2016). Most researchers suggest that a higher SES would have a positive effect on the development of EF, while a lower SES would be associated with poorer executive performance (Johnson et al., 2016).

Although the impact of SES on executive development is relatively well known, studies are mainly conducted in more economically developed countries (Johnson et al., 2016). However, poverty and social inequality contexts are more pronounced in low- and middle-income countries (United Nations Development Programme [UNDP], 2019). Brazil is the fifth largest and sixth most populous country of the world, characterized by a remarkable cultural variability and socioeconomic inequality. Currently, Brazil is considered a middle-income country and presents the 2nd highest income inequality in the world (United Nations Development Programme [UNDP], 2019). This economic gap reveals disparities in key elements of human development such as health and education. In the Brazilian context, guaranteeing access to good education and health services is still strongly dependent on high SES.

In addition, it is important to consider that income distribution is also unequal between the country’s own regions. The South and Southeast regions of Brazil are the most developed of the country, presenting the highest national Human Development Index (HDI) and the highest urban population density. Contrasting, the Northeast region ranks last regarding the HDI. Specifically, the State of Rio Grande do Norte ranks third worst regarding performance in reading, writing and mathematics (Organisation for Economic Co-operation and Development [OECD], 2018). It is important to point out that sociodemographic data from the Brazilian Institute of Geography and Statistics (Instituto Brasileiro de Geografia e Estatística [IBGE], 2010) show that these characteristics are not specific to the Rio Grande do Norte State, but they are shared by most of the other Northeast region states. In addition, Brazilian cities are characterized by a noteworthy socioeconomic variability even within their own boundaries. For example, areas with high HDI levels can be commonly found nearby extremely poor zones (Instituto Brasileiro de Geografia e Estatística [IBGE], 2010).

In this context, it is important to note that 22.6% of children and adolescents between 0 and 14 years of age live in extreme poverty in Brazil. This corresponds to 9.4 million minors with monthly per capita income below or equal to a quarter of the Brazilian minimum wage (Instituto Brasileiro de Geografia e Estatística [IBGE], 2017). This rate is even more expressive in the Northeast region, where the percentage of children in extreme poverty reaches 36.3%. It is necessary to emphasize that poverty in childhood and adolescence goes beyond the lack of money and must take into account other factors that influence a lower quality of life. Considering the fact that access to good education and health in Brazil is strongly associated with a higher SES, children in poverty situation are more susceptible to experiencing worse health conditions, more developmental delays, less school achievements, and more behavioral and emotional issues than their more favored peers (Johnson et al., 2016; Berthelsen et al., 2017).

Brazilian studies have shown differences in EF between children from different geopolitical regions (Hazin et al., 2016), from urban and rural backgrounds (Santos and Bueno, 2003; Santos et al., 2005), and even between children living in the same city but with different SES (Magalhães et al., 2016). However, to the best of our knowledge, studies that considered at least the three basic executive components are scarce and no study proposed so far the analysis of the structure and organization of EF in Brazilian children (Guerra et al., 2020b). Therefore, the main objective of this study was to investigate the developmental trajectories of the basic components of EF: inhibition, cognitive flexibility (including shifting), WM (including updating); and one more complex component: planning. Also, this study aimed to assess the potential effects of two demographic factors (gender and SES) in the development trajectories.

The study was carried out with 7- to 12-year-old children from the Northeast region of Brazil using a battery of performance-based EF tests specially designed for children. We expected (1) an improvement in performance of children between the ages of 7 and 12 years in EF tasks of the different assessed domains (Lehto et al., 2003; Brocki and Bohlin, 2004; Diamond, 2013). We expected progress in EF skills to be evident in executive tests. Regarding the structure and organization of EF, we expected to (2) find a 4-factor structure grouping the tests according to its theoretical assumption. We also expected weak but significant correlations between results of tasks evaluating the same EF if compared to results of tasks that evaluate other executive components (Miyake et al., 2000; Lehto et al., 2003; Bellaj et al., 2015). Considering the relative consensus on the effects of demographic and contextual variables on the development of EF in school-aged children, we expected (3) a positive effect of higher socioeconomic status on executive performance (Magalhães et al., 2016 - Brazilian study; Sbicigo et al., 2013; Noble et al., 2015; Shayer et al., 2015), and (4) a non-significant effect of gender on executive performance (Hazin et al., 2016; Magalhães et al., 2016 - Brazilian studies).

## Materials and Methods

### Participants

A total of 230 Brazilian children from the cities of Natal, Parnamirim and Elói de Souza in the Rio Grande do Norte state participated in the study. The children were aged between 7 and 12 years. The sample was divided into six age groups and each group was composed of approximately 40 children homogeneously distributed by gender and type of school. The study was conducted in 14 public and private schools in the period between 2018 and 2019. The data were collected in four private and four public schools in Natal, four public schools in Parnamirim and two public schools in Elói de Souza.

The research was carried out in accordance with the ethics requirements of the Federal University of Rio Grande do Norte under the code 48383715.1.0000.5537. Participants were selected based on the following inclusion criteria: a) signing of the informed consent form by parents and/or legal guardians; b) regular registration in public or private school; c) absence of a history of developmental, neurological or psychiatric disorders; d) absence of uncorrected sensory alterations; and e) scaled score equal or higher than seven points in the Wechsler Intelligence Scale for Children (WISC-IV) Matrix Reasoning and Vocabulary sub-tests. The selection of participants was carried out in collaboration with the coordinators and teachers of each institution. A total of 264 signed informed consent form were collected and 244 children and adolescents were submitted to the application of the WISC-IV Vocabulary and Matrix Reasoning sub-tests. Fourteen of the participants scored below seven in one of the subtests and, therefore, were excluded from the sample. Table 1 shows the characteristics of the sample population.

**TABLE 1:** Sample characteristics.

	Gender				Type of school				IQ subtests			
	F		M		Public		Private		Vocabulary		Matrix reasoning	
	N	%	N	%	N	%	N	%	M	SD	M	SD
**7 (*n* = 37)**	19	16.38	18	15.78	24	20.68	13	11.40	11.44	2.59	10.56	2.19
**8 (*n* = 41)**	18	15.52	23	20.17	18	15.51	23	20.17	11.10	3.27	11.1	3.12
**9 (*n* = 34)**	18	15.38	16	14.03	17	14.66	17	14.91	11.59	2.53	10.74	3.26
**10 (*n* = 46)**	25	21,55	21	18.42	23	19.83	23	20.17	11.11	2.73	9.82	3.33
**11 (*n* = 39)**	20	17.24	19	16.66	19	16.38	20	17.54	11.73	3.27	9.91	3.09
**12 (*n* = 33)**	16	13.79	17	14.91	15	12.93	18	15.79	11.71	2.89	9.50	2.90
**Total (*n* = 230)**	116	100	114	100	116	100	114	100	11.42	2.88	10.26	3.04

*The values in bold correspond to the significant values.*

### Materials

**FIGURE 1:**
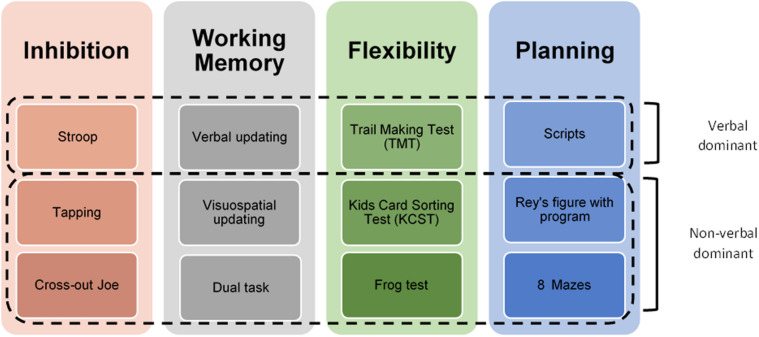
Overview of the CEF-B.

The EF were assessed through the Child Executive Functions Battery (CEF-B). It consists of a set of 12 performance-based tests for the neuropsychological assessment of EF (Figure 1), aimed at children and adolescents between 6 and 16 years old (Roy et al., 2020). The battery is based on a child-centered theoretical model and assesses the main executive processes: inhibition, flexibility, working memory, and planning (Diamond, 2013). It comprises new experimental tasks and tests that already exist in the international literature but have been modified or expanded to better attend child patients.

Given the shortage of EF test batteries based on specific theoretical models in Brazil, CEF-B was adapted to the Brazilian context (Guerra et al., 2020a). Psychometric data of this version indicated good validity and reliability properties (Guerra et al., considered for publication). Also, preliminary evidence of validity of the French version has been published regarding the Stroop test (Roy et al., 2018), and studies with different clinical groups, such as neurofibromatosis type 1 – NF1 (Roy et al., 2010, 2014; Remigereau et al., 2018), parietal temporal and frontal epilepsy (Charbonnier et al., 2011) and brain tumors (Roche et al., 2018). These initial data indicate a good sensitivity of the battery for the evaluation of EF in children.

Table 2 presents a brief description of the tests that compose the CEF-B. The order of application of the tests that integrate the protocol was defined in a systematic and pseudo-random manner, alternating the executive skills investigated and their verbal/non-verbal nature. The purpose of this order is to control the influence of basic processes on executive performance, as well as to have usable tests in case of communication, visuospatial or gestual disorders (Roy, 2015). In order to limit measurement errors, the variables of CEF-B were designed to modulate the executive load involved in some multicomposite tests. This approach consists in providing “control” conditions which are supposed to be less demanding on executive processes (as in subtracting the Trails A score from Trails B score to “isolate” the contribution of executive abilities in the Trail Making Test; Arbuthnott and Frank, 2000).

**TABLE 2:** Brief description of the tests and variables used in the study.

EF	Test	Variables	Brief description of the tests	Proposals for measurement errors and methodological biases control
Inhibitory Control	Stroop	Interference Time	This version is divided in three subtasks: ‘Naming’, ‘Reading’ and ‘Interference’. Three colors are used (blue, red and green) and before the administration of each part, a training phase is performed composed by 10 items in order to verify the children’s understanding of the task. The test proposes 100 items per subtask, divided into 10 lines of 10 stimuli on a white paper in landscape format; the child is instructed to complete each subtask as quickly as possible (timed event) and by committing the least possible mistakes. The interference time score corresponds to the subtraction of the time in seconds of part C (interference) minus part A (naming). Similarly, the interference errors score corresponds to the subtraction of the errors (corrected and uncorrected) of part C minus part A.	-Preliminary control conditions (naming and reading) -Unlimited time, no correction to every mistake made, consideration of time and errors
		Interference Errors		
	Tapping	Go/No-Go Time	It was designed to preferentially evaluate the inhibitory abilities of a predominant or automatic motor response, including a Go/No-Go component and a conflictual conditioning component. After a first phase of “simple conditioning” where the child must repeat the motor action produced by the examiner (tapping once or twice with the index on the table), the child must inhibit this pattern of automated response by no longer reacting when the examiner taps twice (“Go/No-Go” component). In a third and final phase (called “Conflict”), the conditioning becomes antagonistic in the sense that the child must be able to do the opposite of the initial phase (typing twice when the examiner taps once and vice versa), while incorporating a new no-go condition (do nothing when the examiner is tapping with two fingers). The last and most difficult condition therefore imposes both the inhibition of the previously learned response pattern and the automatic “echo” response, and the adaptation to a new no-go component. Each of these three conditions includes a series of 30 items, with sequences varying randomly from one condition to another, to avoid any form of learning. The event is timed and the child’s mistakes are recorded at each phase of the event. To calculate the Go/no go time variable, a subtraction of the time (in seconds) of part B (Go/no go) minus time (in seconds) of part A (simple conditioning) must be made. For the calculation of the Time Conflict, the time (in seconds) of part C is subtracted from the time (in seconds) of part A. The same logic is applied for Go/No-Go and Conflict errors, where both corrected and uncorrected errors are considered.	-Preliminary phase of simple conditioning (repeat a motor action in echo)
		Go/No-Go Errors		
		Conflict Time		
		Conflict Errors		
	Cross-out Joe	Time	It is a test of identification and crossing-out of a visual target among a set of morphologically close distractors, in order to approach the inhibitory control capabilities (inhibition of distractors, selective attention) and sustained attention. The child must cross-out the Joe character from a set of other characters. To verify the understanding of the task, the child performs a training phase with the examiner. The material of the test is composed by two white sheets in A3 format and portrait orientation, on which two series (A and B) of 240 items (16 lines of 15 characters) are randomly distributed. The target (Joe) appears unpredictably for the child, once every five characters, adding up to a total of 48 identifiable targets among 192 distractors (per sheet). This is a timed task in which the child is instructed to work as quickly as possible but also as accurately as possible. The time variable is calculated by the sum of the execution time (in seconds) for series A and B. The error variable is obtained by the sum of the number of omissions (“Joe” forgotten) in series A and B, and the number of false alarms (character other than “Joe” wrongly crossed out) in series A and B.	-Evaluate inhibition in a long-term task
		Speed		
WM	Visuospatial updating	Baseline	It evaluates the updating dimension of the visuospatial capacity of updating. It involves mimicking a researcher as they tap a sequence of up to 10 identical spatially separated blocks. The task consists of two steps: 1- Baseline ‘visuospatial span’: aims to globally evaluate the ability to remember the locations touched by the examiner. 1 point is awarded per recalled location belonging to the series presented. The minimum score is therefore 0 and the maximum score is 30 (5 items of 6 locations). This score will determine the number of locations (n) to be recalled in the updating step. - Score lower than 15: the updating step will not be performed. In this situation, the ability to maintain the information in the short term is considered insufficient for a reliable result. - Score between 15 and 20: the number of locations to be recalled is set at 3 (*n* = 3). When presenting a series of locations of variable length, the child will always only have to return the last 3. - Score higher than 20: the number of locations to be recalled is set to 4 (*n* = 4). When presenting a series of locations of variable length, the child will always have to recall the last 4. 2- Updating: the last three or four items of a given sequence must be recalled. The sequence comprises sets of variable length, from which they must then sequentially recall a specific number of recent elements. There are three types of items: items requiring 3 updates (R3, the number of locations presented is equal to *n* + 3), items requiring 2 updates (R2, the number of locations presented is equal to *n* + 2) and items requiring 0 updates (R0) where all presented locations must be recalled. The recall must be in order and one point is counted for each correct location. The performance score is calculated by sum of points for items without updating and items implying 2 and 3 updates. The task starts with 3 tests to make sure that the setpoint is understood.	-Task adjusted to span capacities. -Variation in the amount of information to be updated to control the executive load (contrasted with items where no update is required)
		Performance score		
	Visuospatial Updating	Baseline	It evaluates the updating dimension of the verbal capacity of updating. Participants are presented with sets of letters of variable length, from which they must then sequentially recall a specific number of recent elements. The task consists of two steps: 1- Baseline ‘verbal span’: aims to globally evaluate the ability to remember the letters recited by the examiner. 1 point is awarded per recalled letter belonging to the series presented. The minimum score is therefore 0 and the maximum score is 30 (5 items of 6 letters). This score will determine the number of letters (n) to be recalled in the updating step. - Score lower than 18: the updating step will not be performed. In this situation, the ability to maintain the information in the short term is considered insufficient for a reliable result. - Score between 18 and 25: the number of letters to be recalled is set at 3 (*n* = 3). When presenting a series of letters of variable length, the child will always only have to return the last 3. - Score higher than 25: the number of letters to be recalled is set to 4 (*n* = 4). When presenting a series of letters of variable length, the child will always have to recall the last 4. 2- Updating: the last three or four items of a given sequence must be recalled. There are three types of items: items requiring 3 updates (R3, the number of letters presented is equal to *n* + 3), items requiring 2 updates (R2, the number of letters presented is equal to *n* + 2) and items requiring 0 updates (R0) where all presented letters must be recalled. The recall must be in order and one point is counted for each correct location. The performance score is calculated by sum of points for items without updating and items implying 2 and 3 updates. The task starts with 3 tests to make sure that the setpoint is understood.	
		Performance score		
	Dual task	Span score	Dual task paradigms involve first performing two tasks separately and then simultaneously. The difference in performance between each separate task and the dual-task condition provides an indicator of the dual-task capability. For this purpose, four subtasks are performed with a duration of one minute and 30 seconds: 1 - digits span (baseline): establishment of ‘span’ (baseline), which corresponds to the number of digits in the last series where there have been at least two successes, that is, for the extension before the interruption; 2 - span task (simple condition): after the establishment of the baseline ‘span’, sequences of the same length are presented for one minute and 30 s; 3- cancelation task: the child is presented with a sheet containing disorderly arranged clown heads connected by a line. For one minute and 30 s the child should draw an X on the clown heads they find on the sheet, following the line; 4- double condition: at this stage, the child is asked to perform the two previous activities simultaneously: make an X on all the clowns’ heads and, at the same time, repeat the series of digits presented by the psychologist according to the baseline established for the “Span” task, for one minute and 30 s. To calculate the variable span score are considered: 1- Number of series correctly recalled in order (EnS) and total number of series proposed (EpS) during the single condition and 2- Number of series correctly recalled in order (EnD) and total number of series proposed (EpD) during the double condition. These indicators allow us to calculate the Span score, which corresponds to the measure of efficiency in double condition compared to the single condition (retention rate): (Pd/Ps)*100 with Ps = EnS/EpS; Pd = EnD/EpD. On the other hand to calculate the Clowns score, the total number of clowns correctly crossed out by the child during the 1 min 30 in both simple (CnS) and double (CnD) condition is needed. Following the same logic of the maintenance rate and efficiency of the double condition in relation to the simple, the computation of the clown score is: (CnD/CnS)*100. In addition to these scores, it is also possible to calculate a Score Mu that expresses the child’s effectiveness in relation to a simple task. The calculation is done by: (Span score + Clowns score)/2	- Preliminary execution of both tasks individually -Task adjusted to span capacities
		Clowns score		
		Score Mu		
Flexibility	Trail Making Test	Flexibility Index	This version is an adaptation of the TMT, which aims to evaluate the ability to alternate the attention focus between sets of stimuli. Unlike the original version, this task consists of three subtasks: subtask ‘A Numbers’, subtask ‘A Letters’ and subtask ‘B Numbers and Letters’. In the first subtask (’A Numbers’) the child is asked to connect numbers (1-20) in ascending order. In subtask 2 (’A Letters’) the child must connect the letters in alphabetical order (A-T) and finally, in subtask 3 (’B Numbers and Letters’) the child must connect letters and numbers alternately while following the alphabetical order and ascending numerical order. A flexibility index is calculated and consists of: (time part B - (time part A numbers + time part A letters)/2) Alternation errors in part B (failure to respect the alternation between a number and a letter), if the subject for example links C to D by omitting the number 4 or links 5 to 6 by omitting E) is also considered.	- Control of numerical and alphabetical chain mastery, visual exploration and perceptual-motor skills in two preliminary parts (numbers then letters, respectively)
		Alternance Errors		
	Kids Card Sorting Test	Time	In this test, the child is required to combine a series of 48 response cards with one of four target cards. Each response card can be combined according to its color, shape and number, and the child must guess what is the combination rule based solely on the evaluators feedback (‘yes’ or ‘no’). After six correct answers, the combination rule is changed. In this version, the child is informed of the three possible classifications beforehand. The time variable is obtained through the time of realization in seconds. The number of perseverations consists of the repetition of an error or continuation of a classification mode when a “no” was formulated for the item just before.	-Only cards that are unambiguous regarding the pairing with the target cards are used - The rules are presented to the child, which reduces the possibility of not understanding the categories
		Perseverations		
	Frog test	Time	This task evaluates the abstraction and deduction capacities of operating rules, which require cognitive flexibility. The child needs to deduce the rules of movement of a frog that moves on several water lilies around a pond according to several logical displacement rules, and to adapt to the actions of the frog, which changes the rule of movement without warning. It is composed by 70 successive cards and in each card there are 10 water lilies (in the middle of a pond, drawn on an A4 sheet in landscape format), numbered from 1 to 10. From one card to another, the frog changes position according to a logical movement rule: the child must therefore anticipate the position of the frog on the next map. Ten rules of displacement are to be discovered by the end of the test, which are not based on mathematical reasoning. The changing of the rule is unpredictable and occurs in a pseudo-random manner every 4 to 9 attempts. The rules of displacement are: -1 (*n* = 6); + 1 (*n* = 5); -2 (*n* = 7); -1 (*n* = 8); High low (*n* = 7); + 1 (*n* = 4); + 2/-2 (*n* = 9); *Status quo* (*n* = 8); + 1 (*n* = 6) and Left-right (*n* = 9). The time variable is obtained through the time of realization in seconds and the score variable consists of the number of correct answers made by the child.	- Random and variable rule change to make the test less predictable
		Score		
Planning	8 Mazes	Completed	This test consists of 8 mazes of increasing difficulty presented on A4 sheets (mazes 1 to 7) or on A3 sheets (maze 8). For each maze, a small dinosaur indicates the starting point, a dinosaur that will have to be lead out of the maze. The exit is marked with a “Exit” flag. The test requires the subjects to draw, with a pencil, the path leading out from the starting point to the exit point, trying not to get into dead-end paths. A maze is considered complete (Completed variable) when the child reaches the end flag in a time less than 240 s. The total time of completion indicates the child’s ability to anticipate and execute the “plan” for solving the maze. The total time variable consists of the average time (in seconds) taken to perform the completed labyrinths. However, if the number of labyrinths presented is less than 6, this average time cannot be calculated. Concerning the impasses, an impasse is counted each time the child goes down a dead-end street and tries to turn back (when he or she realizes it). Specifically, dead ends refer to the drawing of a line that clearly crosses an “imaginary” line that connects each of the two sides of a dead-end street. The impasses variable is calculated among completed mazes and weighted by the number of possible impasses. It is therefore a proportion: the closer it is to 1, the more the child has engaged in all the possible dead ends.	-Consider time and error for the score
		Total time		
		Impasses		
	Rey Osterrieth Complex Figure	Planning Index	In this version, in addition to the traditional copy of the figure – formulation condition –we added the realization of execution condition. In this second part the child progressively reproduced the figure on the basis of successive and progressive cues in which each new group of elements was represented in a distinct color. Children were not informed that they were creating a larger figure from its components. Each cue was presented on a new, separate sheet and children were systematically asked to continue their drawings by including the new colored group of elements. The order of these cues was (1) central rectangle; (2) central diagonals; (3) two major horizontal and vertical lines and upper and major right triangles; (4) diamond at the end of the major triangle, vertical line in major right triangle, lower left square and lower cross attached to vertical midline below rectangle, and upper left cross outside of rectangle; and (5) minor rectangle with diagonals, horizontal lines in upper left corner of central rectangle, vertical line in upper right corner of central rectangle, upper right circle, and lower right oblique lines. The Copy Score (C) of the formulation condition is rated as conventionally assessed on 36 points (accuracy and location considered) and a Program Score (P) of the execution condition is also rated based on the same scoring principles (each of the 18 items rated on 2 points, maximum total on 36 points). From these scores a Planning Index (PI) is calculated. It consists of a proportion where PI: (P/C) *100. The higher the PI, the more the child benefits from the help provided by the program, and the more the difficulties identified in spontaneous copying can be attributed to a problem in planning and organizational strategies.	-Measurement of the facilitating effect of copying with the program in contrast to spontaneous copying -Rigorous and objective instructions for the evaluation of the precision and location of the figure elements
	Scripts	Time	The Scripts task is composed of a series of sequential everyday life actions. These schemes form conceptual units that allow the individual to be prepared to think and act in specific contexts. The scripts proposed in this task are: 1 - Take a shower; 2 - Prepare the backpack to go to school; 3 - Do the shopping at the supermarket. For each script there are actions that the child must put in order to build a coherent script according to the given title. Among the cards provided, two of the envelopes (Envelope 1 - Take a Shower and Envelope 2 - Prepare School Bag) contain actions considered as intrusive, i.e., actions that are not related to the script. The child must justify the order of the arrangements, including the intrusions, explaining his or her behavior in relation to them. It is important to note that at no circumstances is the child informed that there are intrusions in the scripts, and whenever the child questions these actions, the examiner answers without giving the child the impression that he or she has the right to reject or accept the intrusion. The time variable is obtained through the sum of time of realization in seconds of the 3 scripts. The total of 3 scripts is composed by 22 actions. The sequence errors are considered as poorly placed items in the expected order of the actions. Thus, the variable sequence errors can vary from 0 to 22. The Intruders variable is calculated by the number of semantically implausible intruders placed within the target action sequence for the two scripts with intruders (by the candies, break the eggs for the “Shower” script and prepare the salad, search for the shells for the “Schoolbag” scripts). Semantically plausible intruders (swimming for the “shower” script; sitting for the “schoolbag” script) placed in the target-action sequence are not considered for the Intruder variable. They are considered in a qualitative way as a weakness of internal coherence in the representation of the script.	-New task created to evaluate the child’s ability to anticipate the order necessary for the execution of a daily action
		Sequence errors		
		Intruders		

Regarding the evaluation of SES indicators, a questionnaire for parents was created to retrieve information on the type of school (public or private) the child is enrolled, family income, level of education and profession of the parents. All variables were initially considered for analysis. However, the only variable with no missing values was ‘type of school’. Thus, we opted to use it as the sole indicator of SES, because the existence of missing values in any independent variable impairs the analysis of the other variables. In addition, we verified that ‘type of school’ was highly correlated with parents’ level of study and family income (*r* = 0.675 to .750; *p* < 0.001), assuring the representativeness of the measure. It is important to highlight that in the Brazilian context, guaranteeing access to good education and health services is strongly dependent on high SES. In fact, children from higher SES attend private schools and children from more disadvantaged contexts attend public schools.

### Procedure

All participants were individually evaluated in a quiet room in their school or home environment. Depending on the age of the child, two or three assessment sessions were needed with a duration of approximately 30–40 min each. All the tests were administered by trained neuropsychologists using standardized instructions. The tests were systematically presented in the same order: 8 Mazes, Stroop, Visuospatial updating, Scripts and Tapping tasks were proposed at the first session and the Rey Complex Figure, Trail Making Test, Dual task, Kids Card Sorting test, Cross-out Joe, Verbal updating test and Frog test were proposed during the second session. An additional session was conducted with younger children. In this case, each session consisted of 4 tests per session, in the aforementioned order.

### Statistical Analyses

The scores obtained in the various tests were subjected to descriptive and inferential statistical analysis. The Kolmogorov-Smirnov test revealed that the data is not normally distributed. Given the importance of examining the effects of interactions between dependent variables (age, gender, type of school), we opted for carrying out a data normalization process (Soloman and Sawilowsky, 2009) and using parametric statistical tools. For this end, we used three-way analysis of variance (ANOVA) followed by an assessment of the weight of the effect by means of partial eta squared. Through this procedure we were able to study both the developmental and differential aspects of each executive process. When the effects were significant, we used the Tukey HSD *post hoc* test to refine the results.

In order to preliminarily analyze the structure of the executive development in our sample and to examine the theoretical grouping considered in the CEF-B, the variables were subjected to a correlation and exploratory factor analysis. Given that the battery comprises 12 tasks that are often represented by more than one score variable, carrying out a factor analysis with 36 variables and 230 subjects would not be the correct approach. Therefore, we decided to reduce the total amount of variables to 12 (one per task) in order to provide a more trustworthy analysis. However, as highlighted in the literature, selecting executive measures is a complex undertaking because the strategy adopted to select a score might influence the results. For example, a child who gives a very fast but random response to a task would obtain an excellent time score, although his performance is far from optimal. Given the wide variety of measurements proposed in the battery, different efficiency scores were selected for each task based on their particularities. For inhibition tasks, we used the Inverse Efficiency Score (IES), which is an approach that takes into consideration both time and accuracy measures (Vandierendonck, 2017). Since the updating tasks (verbal and visuospatial - performance score) and the planning index of the figure of Rey do not present measures of time, the IES was not calculated and the aforementioned indices were used instead (see Table 2). In addition, the score Mu (Della Sala et al., 2010) was chosen for the Dual Task, since it can also be considered a measure of efficiency and it is a classic approach for dual task paradigms. For all flexibility tasks and the other planning tasks (8 Mazes and Scripts) the most representative measures of the executive charge of the task were used. In the case of flexibility tasks, time measurements were chosen as most representative since the amount of errors is always low. For 8 Mazes, we used the weighted errors as representative measures since the available time to complete the labyrinths is limited. The error measure was also chosen for the scripts task, given the implication of planning and anticipation in the correct sorting of actions. Horn’s parallel analysis was used to determine the number of components of factor analysis (Horn, 1965). The equamax extraction method was used beforehand to assure an orthogonal rotation. All statistical analyses were performed using SPSS v.20.0 (IBM Corp. Released, 2011, Armonk, NY, United States).

## Results

### Age, Gender and SES Effects

To test the effect of age, gender and type of school on all EF measures, we conducted a set of three-way ANOVAs. The descriptive data and a summary of the main effects are shown in Tables 3, 4. *Post hoc* analyses and trend analyses for age affect are described in Table 5. In the following sections, we describe the results of the analysis by EF process. Significant results showed a moderate to high effect size for age and moderate effect size for gender and type of school, according to Cohen (1992) classification. For all analyses, the significance level for p was set at.05.

**TABLE 3:**
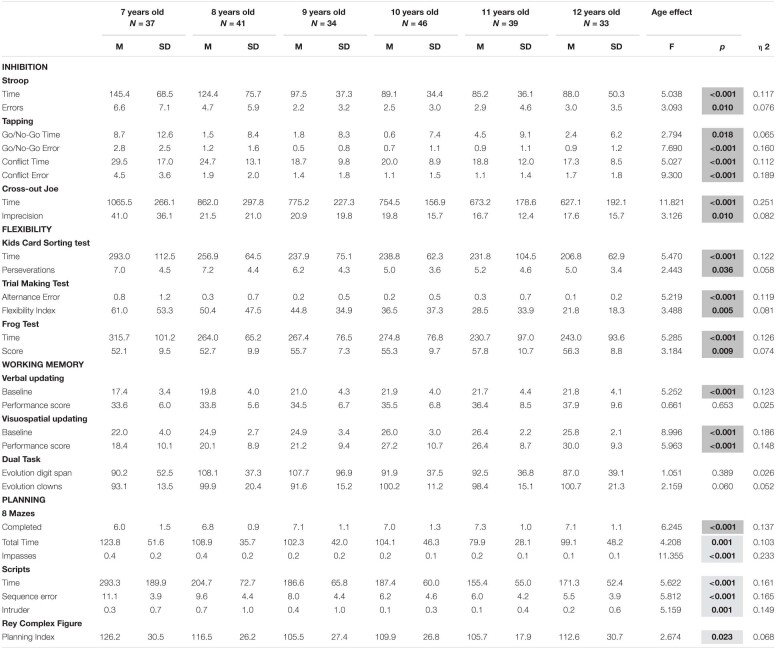
Age effect on EF variables.

*M, Mean; SD, Standard deviation. The values in bold correspond to the significant values.*

**TABLE 4:**
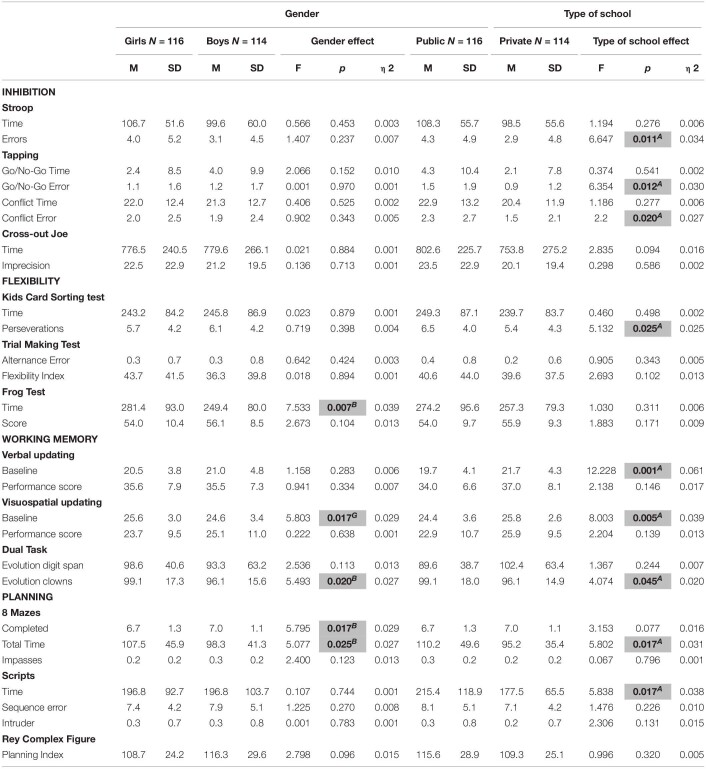
Gender and type of school effect on EF variables.

*M, Mean; SD, Standard deviation; G, Girls had a higher mean value than boys; B, Boys had a higher mean value than girls; A, Private school children had higher mean values than public school children. The values in bold correspond to the significant values.*

**TABLE 5:**
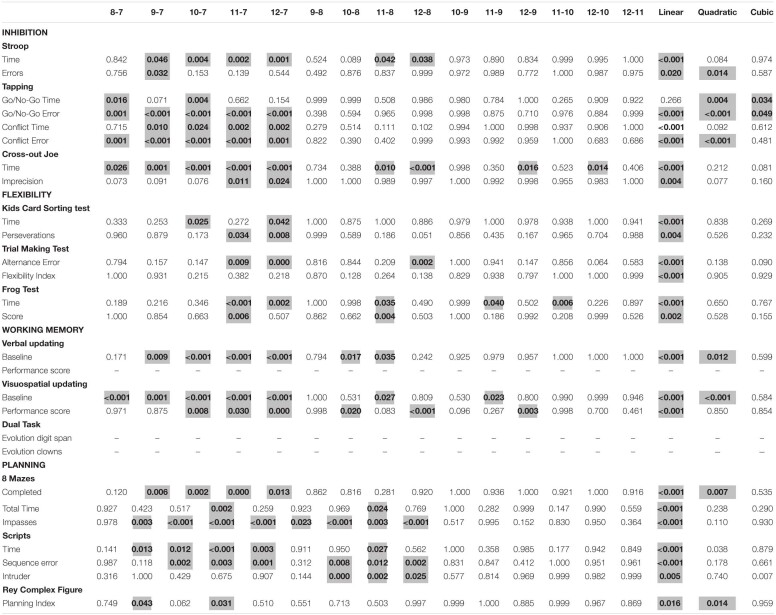
*Post hoc* analyses and trend analyses for age effect.

*The values in bold correspond to the significant values.*

#### Inhibition

The analysis revealed a significant effect of age for all inhibition measures. We found a linear improvement with age in results for all variables, with exception of Tapping Go/No-Go Time variable. In addition, a significant quadratic trend was observed for Tapping test (Conflict Error, Go/No-Go Time and Error variables) and Stroop (Errors variable). Also, a significant cubic trend was observed for Tapping test (Go/No-Go Time and Error variables). In the other hand, the gender effect was non-significant for all inhibition measures. The age by gender interaction effect was also non-significant. Regarding type of school, results revealed a significant effect for the Stroop test Errors, Tapping Go/No-Go Errors and Tapping Conflict Errors variables. In both cases, private school children performed better than those from public school. Interaction between type of school and gender was significant only for Imprecision (*F*(1) = 8.298, p.004, ηρ2 = 0.046). However, *post hoc* analysis did not show significant differences between groups. In addition, the age by type of school interaction effect was significant for Cross-out Joe Imprecision *F*(5) = 0.298, p.586, ηρ2 = 0.002 and Go/No-Go Errors *F*(5) = 6.354, p.586, ηρ2 = 0.002. For the Cross-out Joe test, *post hoc* analysis showed that 7-year-old children from private schools had worse results than 8 and 12-year old children from the same type of school. Regarding Go/No-Go Errors, 7-year-old children from public school performed poorer than 8, 9, 10 and 12-year-old from the same type of school and 8 to 12-year-old from private schools. The *post hoc* analysis showed that children of 11 years old from private schools performed better than children at the same group age from public schools. In general, the interaction effects between age and type of school showed better results for older children if compared to younger children from the same type of school. In addition, results were better for children from private schools if compared to their public-school peers or younger children from public school.

#### Flexibility

A significant age effect was observed in the totality of flexibility measures. In addition, all results presented a linear trend. A significant effect of gender in favor of boys was found only for Frog test Time *F*(2) = 7.533, p.007, ηρ2 = 0.039. Gender interaction with age and with type of school were non-significant. Regarding type of school, the analysis revealed a significant effect for KCST. Public school children had worse scores than private school children. Age by type of school interaction effect was significant only for TMT Flexibility Index (*F*(5) = 2.479, p.033, ηρ2 = 0.060). *Post hoc* analysis showed that children aged 7 from private school has worse scores than children aged 12 and 10 from public school and 12 years-old children from private school. Also, *post hoc* analyses revealed that 8-year-old from public schools has performed worse than 10 and 12-year-old from the same type of school.

#### Working Memory

A significant age effect was observed for the Verbal (Baseline variable) and Visuospatial updating (both Baseline and Performance score variables). These variables presented a linear trend in the means across age groups. Moreover, the Baseline variable showed a significant quadratic trend for Verbal and Visuospatial updating tasks. Analysis revealed a significant effect of gender in favor of boys for the Visuospatial updating (Baseline), and for the Dual task (Evolution clowns). The age by gender interaction effect was significant only for the Dual task (Evolution clowns). *Post hoc* analysis showed that 12-year-old girls have lower scores than 7 and 9-year-old boys. In addition, interaction between gender and type of school was also significant for the Dual task (Evolution Clowns variable). *Post hoc* analysis revealed that public school girls have lower scores than public and private school boys, and private school girls. Type of school had a significant effect for Dual Task (Evolution clowns), Verbal and Visuospatial updating (Baseline). Public school children had worse performances than private school children. Age by type of school interaction was significant for Verbal updating (Performance score). However, *post hoc* analysis did not show significant differences between groups.

#### Planning

The analysis revealed a significant effect of age for all planning measures. We found a linear improvement with age in results for all variables. In addition, a significant quadratic trend was also observed for 8 Mazes (Completed variable), Script (Time variable) and ROCF (Planning index) tests. A significant cubic trend was also found for the Script test (Intruder variable). A significant effect of gender in favor of boys was found for Mazes (Completed and Total Time variables). In addition, the age by gender interaction effect was non-significant, as well as the gender by type of school interaction. The type of school effect was significant for Mazes Total Time and Scripts Time. In both cases, private school children performed better than those from public school. Furthermore, the age by type of school interaction effect was non-significant.

### Factor Analysis and Correlations Between EF Components

Concerning the exploratory factor analysis, Table 6 presents the factor loadings with equamax rotation. Four factors emerged: tasks known to be related to inhibition (Stroop, Tapping, Cross-out Joe) were represented by the first factor; tasks related to WM (Verbal and Visuospatial updating, Dual task) were explained by the second factor; two flexibility tests (Frog test and KCST) were captured by the third factor; and the other scores related planning (ROCF, Mazes and Scripts), one flexibility task (TMT) and one WM test (visuospatial updating) were represented by the fourth factor. We chose to name Factor 1 as “Inhibition,” Factor 2 as “WM,” Factor 3 as “Flexibility” and Factor 4 as “Planning.” It should be noted that the variables that constitute Factor 4 include measures of both WM (Visuospatial updating) and flexibility (TMT).

**TABLE 6:** Factor analysis pattern matrix for CEF-B tasks.

	Factor loading			
	1 (INH)	2 (WM)	3 (FLEX)	4 (PLAN)
Stroop	0.58			
Tapping	0.66			
Cross-out Joe	0.65			
Trail Making Test				0.55
KCST			0.76	
Frog test			0.82	
Dual task		–0.70		
Verbal updating		0.78		
Visuospatial updating		0.31		–0.61
Mazes				0.64
ROCF				0.49
Scripts				0.75

*Values less than 0.30 were excluded.*

In order to evaluate correlations among CEF-B tasks, we performed several correlation analyses between the scores (Table 7). Results show that inhibition measures are significantly correlated. Similarly, flexibility measures are also significantly correlated. WM tasks present significant correlations between verbal and visuospatial updating; although the dual task did not correlate with them. In addition, planning measures also presented significant correlations, with the exception of ROCF and Mazes.

**TABLE 7:**
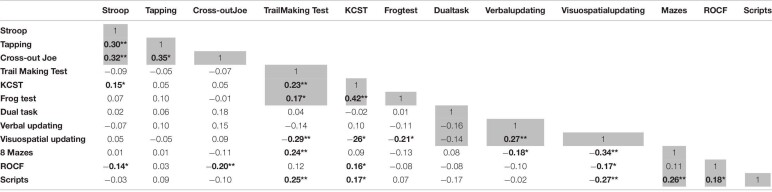
Correlation between CEF-B tasks.

*^∗^ = p<0.05; ^∗∗^ = p<0.01. The values in bold correspond to the significant values.*

## Discussion

The main objective of this study was to investigate the developmental trajectories of four EF: inhibition, cognitive flexibility, WM, and planning. Furthermore, this study aimed to assess the potential effects of two demographic factors (gender and SES) in the development trajectories and present an initial analysis of the structure and organization of EF in Brazilian children.

Regarding the developmental objective, the analyses of the age effect on inhibition measures revealed a linear improvement in results for all inhibition variables. Our results are consistent with those found in other Brazilian versions of Stroop conducted with children between 7 and 10 years old (Charchat-Fichman and Oliveira, 2009) and adolescents between 12 and 14 years (Duncan, 2006), which also presented a linear downward trend with age for time and errors. In addition, the reduction of the Stroop effect with age is also consistent with studies conducted with 7 to 12-year-old children in America (Mexico - Armengol and Méndez, 1999; United States - Adleman et al., 2002; Davidson et al., 2006), Europe (Sweden - Brocki and Bohlin, 2004; France - Roy et al., 2018), Africa (Tunisia - Bellaj et al., 2015) and Asia (China - Xu et al., 2013). This finding supports the idea of an active development of EF during childhood (Best and Miller, 2010). Also, regarding the Tapping test, previous Brazilian studies with similar Go/No-go paradigm also showed an improvement in speed and in the amount of errors committed by the children (Charchat-Fichman and Oliveira, 2009; Salles et al., 2016).

Developmental data on WM revealed a linear and quadratic trend increase on verbal and visuospatial updating tasks for the *Baseline* variable. The quadratic and linear trend for these variables indicates a linear improvement in data until a peak value is reached, which suggests a change in data behavior. In the case of the verbal and visuospatial baselines, this developmental peak seems to occur at the age of 11. Also, we found a significant linear trend for the variable *Performance score* of visuospatial updating. Our results are consistent with other Brazilian studies that used different paradigms, but aimed to evaluate the updating component through a verbal or visuospatial task (Santos et al., 2005; Wechsler, 2013). They are also in consonance with findings from the international literature that aimed to evaluate verbal and visuospatial WM skills (Best and Miller, 2010). The review carried out by these authors regarding WM and updating suggests that the developmental trajectories of these functions are linear from preschool through adolescence. It is important to note that the significant effect of age was found only for the visuospatial component of the updating task. One possible explanation for this result is associated with a poorer baseline performance found for the verbal task if compared to its visuospatial version. In fact, the baseline represents a cut-off point for performing the update task, and consists of retaining the maximum number of items presented by the examiner. The authors established that the cut-off point for the visuospatial component should be 15 points (out of a maximum of 30), while for the verbal component the cut-off point should be 18 (out of a maximum of 30) (Roy et al., 2020). These different threshold values were defined based on the studies by Yue et al. (2008), which indicated a better performance in verbal short-term memory skills than in spatial ones.

However, these assumptions did not seem to be pertinent in the Brazilian context, since we find poorer verbal baseline performance if compared to visuospatial baseline performance, especially in public school children. In fact, 53 children scored less than 18 in the verbal updating task while only 8 children did so in the visuospatial updating task. If we set the cut-off point to 15, this number reduces to 18 children for the verbal updating task, and only one child for the visuospatial component (as currently calculated). Thus, only the most performant children had their scores accounted for at the updating stage of the test. Therefore, it seems appropriate to consider an adjustment of the cut-off point of the verbal baseline in order to match it with the visuospatial version.

Furthermore, the type of school factor also plays an important role in the interpretation of these results. They show that the scores of children from private schools improve with age, while the performance of children from public schools tends to be stable. To support this finding, we conducted complementary comparison analysis to investigate possible differences in the performance of children from private and public schools on the Vocabulary (verbal competences) and Matrix reasoning (spatial perception, visual and abstract processing) subtests of the Wechsler Intelligence Scale for Children (WISC-IV). We found a significant difference for Vocabulary (*p*<0.001), with public school children performing poorer than private school children, while for Matrix reasoning the comparisons were non-significant (*p*<0.103). This result is consistent with the current literature that reports the impact of SES on cognition, especially language skills. Numerous studies show that the verbal abilities of children from disadvantaged socio-economic backgrounds are poorer than those of children from privileged backgrounds (Johnson et al., 2016; Merz et al., 2019). These results represent a measurement bias and an adaptation issue for the Brazilian context that must be reconsidered in future studies.

In addition to verbal and visuospatial updating tasks, we proposed the evaluation of WM in double condition. Dual-task paradigms involve performing two tasks separately first, and then simultaneously. The difference in performance between each separate task and the dual-task condition provides an indicator of dual-task ability (Della Sala et al., 2010). Thus, two variables were used to access WM skills in double condition (Evolution digit span and Evolution clowns) and no significant age effect was found. The absence of this effect can be explained by the fact that this variable is adjusted to age. In fact, the first part of the Dual task consists of defining the child’s baseline through a span score. This baseline represents the level of difficulty of the task, which is determined by the child abilities’ and corresponds to the maximum number of sequential digits that the child can remember without committing an error. Thus, difficulty levels differ according to individual variations, but also according to age given the improvement in auditory span memory (Baddeley et al., 1997). These particularities of the Dual task may have minimized a potential age effect on this task.

Regarding flexibility measures, analyses of the age effect revealed a significant difference between groups and a linear trend for all tasks. For the KCST, older children tend to complete more categories and be more agile in performing the task, as shown in previous studies performed with the Wisconsin Card Sorting test (Chelune and Baer, 1986; Heaton et al., 2007). Concerning both variables of the Frog test, results revealed a significant overall improvement with age, as evidenced in similar tasks (Burgess and Shallice, 1997). In general, behavioral profiles of the performance of Brazilian children in flexibility tasks show that the increase is more evident when comparing performances between the oldest (10–12 years) and the youngest children. Older children were faster and more precise in performing the task, which is used worldwide and has many variants.

Concerning planning skills, developmental data revealed a linear increase in performance on the Mazes, ROCF and Scripts tasks. Additionally, a quadratic (Mazes - *Completed;* Scripts - *Time*; ROCF – *Planning Index*) and cubic (Scripts – *Intruder* variable) trend was also observed, revealing a propensity to spurts or developmental peaks. Performance profiles of Brazilian children in planning tasks show that the strategies used to complete the test depend on its nature and on the child’s age. For example, concerning the ROCF planning index, two peaks of improvement seem to emerge at 9 and 12 years of age. This index presents a significant decreasing score from 9 to 11 in comparison to 7 years old. At the age of 12, in the early adolescence, an increase in the planning index associated with the benefits of the guided copy (execution) step is identified. This change in data behavior can be related to the developmental changes on EF typically observed in adolescence, but should be better explored in the continuity of this study. On the other hand, Script and 8 Mazes tasks seem to present gradual improvement profiles with differences between the ages of 9 to 12 and the age group of 7. Studies that used different assessment paradigms, but aimed to evaluate planning skills for visuospatial and verbal paradigms also showed a similar improvement profile (Wechsler, 2002; Marquet-Doléac et al., 2010; Rey, 2010). These studies showed that planning skills improved with age, although they suggest different peaks in maturity, that occur mainly in adolescence. In this sense, our data would only represent the early maturation of this function at school age and should be extended to include the adolescent population.

Regarding the effect of other demographic variables studied, our results showed that the comparison between gender and executive measures did not reveal a significant difference for Brazilian children, except for the Visuospatial updating task, Dual Task, Frog test and 8 mazes. These findings are consistent with data in the literature which show no or little gender effects on executive development (Anderson, 2002; Brocki and Bohlin, 2004; Lee et al., 2013; Xu et al., 2013). Our results are also consistent with Brazilian data which showed that this factor has little influence on EF (Hazin et al., 2016; Magalhães et al., 2016). These results support the idea of a global performance equivalence between girls and boys regarding EF (Lehto et al., 2003; Brocki and Bohlin, 2004). However, it should be noted that the significant results are all in favor of boys, although they were found in only four of the 12 tasks. In addition, interactions between gender and type of school for Dual task (Evolution clowns) revealed that public school girls have lower scores than public and private school boys, and private school girls. These gender results show that girls in vulnerable situations are at a disadvantage if compared to boys, which was previously demonstrated in international reports and studies (Qadir et al., 2011).

Regarding the effect of SES on executive development, we found a significant effect on 8 tasks (Stroop – *Error*; Tapping – *Go/No-Go Error* and *Conflict Error*; KCST – perseverations; Verbal and Visuospatial updating - *Baseline*; Dual task – *Evolution clowns*; Scripts – *Time*; Mazes – *Total time*). It is important to mention that all executive components were accounted for and all results were in favor of private school children. Our findings are in accordance with the literature regarding the beneficial effect of a favorable SES on executive development (Johnson et al., 2016; Farah, 2017; Lawson et al., 2017). Additionally, other Brazilian studies have also found differences in favor of higher SES regarding inhibition, WM and decision-making skills (Mata et al., 2013; Hazin et al., 2016; Magalhães et al., 2016; Sallum et al., 2017). Our study also highlights the impact of socioeconomic disparities on the development of cognitive flexibility and planning skills.

An exploratory factorial analysis and several correlation analyses were performed in order to preliminarily analyze the structure of the executive development in our sample and to examine the theoretical grouping considered in the CEF-B. We found a 4-factor structure as described in the theoretical distribution proposed by the battery. However, the organization of the tasks in the components did not correspond to our initial expectations. Factors were named as follows: factor 1 “Inhibition,” factor 2 “WM,” factor 3 “Flexibility” and factor 4 “Planning.” Indeed, factor 1 is similar to the classification originally designed by the authors of CEF-B, and correspond essentially to tasks of inhibition. Similarly, factor 2 corresponds to all WM tests. Indeed, evidences in the literature on the development of EF in children suggest that inhibition and WM are the first components to differentiate (at approximately 5–6 years of age; Diamond, 2013; Lee et al., 2013). This feature may explain the more evident grouping of these factors.

Factor 3 groups two tasks of flexibility. Only the TMT was not comprised by this factor. On the other hand, factor 4 grouped flexibility, WM and planning tasks. There are two main explanations for this result. First, the flexibility and planning components are the last to differentiate themselves according to the developmental logic (Diamond, 2013). Studies show an improvement in flexibility and planning skills from 10 to 12 years and in adolescence (Anderson et al., 2001; Brocki and Bohlin, 2004; Lee et al., 2013). In this sense, it is important to consider that the age limit of 12 years in our sample may have contributed to the clustering planning tasks with other components, since they would only differentiate themselves later in development. In this sense, expanding the sample to include adolescents is essential in order to assure analyses that are more consistent with the development of these complex functions. Another explanation is associated with the fact that flexibility and planning, especially, are complex tasks, and may have been grouped together because of their complexity. Regarding the visuospatial updating task, the association with both the planning and the WM factor may be related to the use of different strategies to recall the last locations touched by the examiner. This aspect can be the object of future investigation in a qualitative way (e.g., by asking the children what strategy they used to recall the locations).

Concerning correlation analysis, inhibition measures were significantly correlated among themselves. Similarly, flexibility measures were also significantly correlated among themselves. However, it should be considered that flexibility tasks also significantly correlate with planning and updating measures. Also, TMT loads with one updating measure and all planning tasks in the factor analysis. These findings also indicate that cognitive flexibility should not be considered as a unitary factor as proposed by Diamond (2013). Literature shows that tasks derived from the WSCT are considered shifting tasks (Morra et al., 2018). In addition, even if the TMT can be considered a global test of executive functioning, it still presents an important shifting component. The test also places high demands on inhibition – by suppressing the strong associations between consecutive numbers or letters – and updating – by remembering the latest number while searching for a letter and vice versa. On the other hand, the Frog test seems to be more associated with the concept of flexibility (Burgess and Shallice, 1997), given the involvement of the ability of abstraction and deduction of operating rules, which primarily requires cognitive flexibility.

Regarding WM tasks, verbal and visuospatial updating were significantly correlated but the dual task did not correlate with them. This finding can be explained by the theoretical differences associated with WM and the concept of updating (Morra et al., 2018). It is worth mentioning that we initially based the categorization of the executive components evaluated in the CEF-B on the theoretical perspective of Diamond (2013). However, this classification presents limitations regarding the theoretical distinction between updating and WM. In fact, the dual task was not loaded into this factor probably because this paradigm is strongly associated with the concept of WM (Logie, 2016) while the tests of verbal and visuospatial updating constitute updating tasks (Morra et al., 2018). Similarly, planning measures were significantly correlated, with the exception of ROCF and Mazes. This finding can also be associated with the fact that this component does not have a unitary dimension. In general, results of the correlation analysis corroborate with the findings of the factorial analysis, which better differentiated inhibition and WM if compared to flexibility and planning. Overall, they are consistent with factorial studies and theoretical modeling in children that consider inhibition, WM and flexibility the main basic components of EF (e.g., Lehto et al., 2003; Diamond, 2013).

There are some limitations to our study that should be addressed in future researches. Firstly, the sample size does not allow normative data to be used in clinical settings and does not allow for generalization to the Brazilian context (see Guerra et al., 2020b for a review). In addition, analyses of the structure and organization of the EF can only be conducted as an initial approach. A series of methodological considerations must be taken into account regarding the factorial analysis. Firstly, our sample was reduced to 150 subjects because children missing one or more measurements needed to be excluded from the sample to meet the method’s requirements. In addition, the entire sample was composed of children with typical development. The lack of children with clinical conditions reduces the discriminating power of the analysis since the variance of measurements in children without pathologies is limited. Finally, the variables selected to be used in the factorial analysis corresponded to the indicators of the best child performance per task. Although it is an interesting approach that favors the best strategy used by the child when performing the task, it would be more interesting to dispose of a vulnerability score which consists of an average of low scores. In this case, however, normative data would be required in addition to clinical data, which was not possible at this stage of the research. Thus, the sample should be expanded considering Brazil’s social disparities and the tests should be submitted to other stages of psychometric validation. In addition, studies comprising different clinical conditions should also be carried out in order to test the sensitivity of the battery and to assure its clinical validity.

To conclude, this study reveals a dynamic developmental progression in all EF assessed by CEF-B tasks in Brazilian children from the northeast region. While gender seem to have little impact on EF development in our sample, the impact of SES on children’s performances confirms the influence of poverty on the development of EF. Thus, the findings of our study highlight the urgent need to design consistent public policies that stimulate children development in vulnerable and disadvantaged populations. In addition, health and education professionals need to consider these differences in the development trajectories of EF and provide stimulation strategies that promote the development of children from unfavorable contexts. Although normative data are still lacking in Brazil, we believe that the next stages of this research will allow a better understanding of the trajectories of EF both in typical and atypical development. Also, these future data will provide clinical neuropsychologists with an improved theoretical basis for child executive development and tools for identifying executive disorders.

## Data Availability Statement

The raw data supporting the conclusions of this article will be made available by the authors, without undue reservation.

## Ethics Statement

The studies involving human participants were reviewed and approved by Comitê Central de Ética em Pesquisa (CEP Central) da Universidade Federal do Rio Grande do Norte (UFRN). Written informed consent to participate in this study was provided by the participants’ legal guardian/next of kin.

## Author Contributions

AG designed the study, collected and screened data, conducted the statistical analysis, interpreted the results, and wrote the manuscript. J-LR screened data and reviewed the manuscript. YG provided advice writing the manuscript. AR, IH, and DL supervised the study design and provided guidance. All authors were involved in critically revising the manuscript, approved the final version, and agreed to be accountable for all aspects of the work.

## Conflict of Interest

The authors declare that the research was conducted in the absence of any commercial or financial relationships that could be construed as a potential conflict of interest.
